# Comparative Study of the Effect of Liraglutide and Donepezil on Learning and Memory in Diazepam-Induced Amnesic Albino Rats

**DOI:** 10.7759/cureus.41495

**Published:** 2023-07-07

**Authors:** Kusum Kumari, Sonu Kumar, Ritesh K Sinha, Mary Sunita Toppo

**Affiliations:** 1 Pharmacology, Rajendra Institute of Medical Sciences, Ranchi, IND; 2 Pharmacology and Therapeutics, Rajendra Institute of Medical Sciences, Ranchi, IND

**Keywords:** amnesia, diazepam, memory, donepezil, liraglutide, dementia

## Abstract

Background

Dementia is an age-related gradual loss of memory that is progressive in nature. Presently, the most common cause of dementia is Alzheimer’s disease (AD), which is treated with donepezil, an anticholinesterase. But it only provides short-term symptomatic improvement. Liraglutide, which is an anti-diabetic drug, stimulates the anti-apoptotic pathway of nerve damage, which helps in regenerating nerve cells; so, it may help in dementia cases. Therefore, this study aimed to explore the effect of liraglutide on learning and memory and to compare its effect with donepezil in diazepam-induced amnesic albino rats.

Methodology

Twenty healthy male Albino rats weighing 150-200 grams were taken and divided into four groups: A, B, C, and D. Group A rats were normal rats, whereas the rats in groups B, C, and D were made amnesic by the intraperitoneal (i.p.) administration of 0.1 mg per kg of diazepam. Immediately after producing amnesia, group B rats received normal saline, group C received liraglutide, and group D received donepezil through the intraperitoneal route as test drugs. Group A rats received only normal saline. The amnesic effect was measured by the escape latency period, which was measured by using a Morris Water Maize (MWM) instrument. Escape latency is the time (in seconds) to locate the platform from the starting point. The amnesic effect is shown by an increase in escape latency and the anti-amnesic effect by a decrease in escape latency. Escape latency was recorded at 0 hr, 1 hr, 2 hr, 3 hr, and 4 hr after test drug administration.

Results

Group B rats showed an increase in escape latency, which shows the amnesic effect of diazepam. When group C and group D amnesic rats were treated with liraglutide and donepezil, respectively, a one-hour after-treatment increase in escape latency was seen but after two hours, both groups showed a decrease in escape latency, which indicates the anti-amnesic effect of both drugs. When groups C and D were compared, and the post-hoc highly significant difference (HSD) test was used, there was no significant difference between the two drugs, although the liraglutide-treated group (C) showed a lower anti-amnesic effect. However, group C showed a significant effect as compared to group B rats (p-value <0.05), which indicates the anti-amnesic property of liraglutide as compared to normal saline.

Conclusion

Liraglutide shows an anti-amnesic property. Since it works by a mechanism different from donepezil, it can be used as add-on therapy with donepezil in dementia patients.

## Introduction

The process of acquiring knowledge and skills from any source is called learning, and retention of that information in the brain is called memory. Dementia is a mental problem with gradual loss of memory, so it mostly affects elderly people. More than 5.5 million people worldwide have dementia and about 60-70% suffer from Alzheimer’s disease (AD) [1].

The most common cause of dementia is AD. Patients with preclinical AD show more memory and learning deficits as compared to those having no evidence of AD [2]. Two major biomarkers are beta-amyloid deposition and the formation of neurofibrillary tangles (NFTs). Donepezil is an anticholinesterase that increases acetylcholine (Ach) levels in the brain and thereby produces improvements in memory in AD patients [3]; however, it only delays the progress of the disease [4]. Till now, AD has not been successfully treated by any drug [5]. The total number of dementia cases is also gradually increasing on a day-to-day basis, hence, there is a great need to develop a novel treatment for AD.

A close association has been found between type 2 diabetes mellitus (DM) and AD. In type 2 DM, insulin resistance increases cerebral glucose concentration, which causes neuronal cell death by oxidative stress or mitochondrial dysfunction [6]. Previously, the brain was thought to be an organ unaffected by insulin but now it has been confirmed that insulin and Insulin receptors are also present in some brain areas [7]. This led scientists to search for new drugs that help regenerate nerve cells or protect them from damage. Recently, evidence shows that liraglutide has a neuro-protective effect so it can be used as a memory enhancer drug [8]. Liraglutide is a novel drug that is used to treat type 2 DM. It is a GLP-1 analog and has 97% amino acid homology with native GLP 1. Its receptors are present in the islets of Langerhans, the gastrointestinal tract (GIT), and the brain. It is given subcutaneously (S.C.) once daily due to its longer half-life (13 hours) [9] due to which it maintains 24-hour glycemic control [10].

Rats were chosen for our study due to their small size and greater sensitivity to most drugs. They are particularly suitable for testing psycho-pharmacological agents because they can be trained properly for various types of work performances including the development of conditioned reflexes [11]. We used diazepam for the induction of amnesia in Albino rats. The objective of this study was to explore the anti-amnesic effect of liraglutide and to compare it with donepezil in diazepam-induced amnesic Albino rats using the Morris water maze (MWM) model.

## Materials and methods

Twenty male Albino rats were randomly divided into four groups (A, B, C, and D) of five rats each. They were kept in their cages at normal temperature and humidity. They were fed with standard laboratory diet and water was also provided to them. Cages were regularly cleaned to prevent infection. The experimental protocol was approved by the Institutional Animal Ethics Committee of RIMS Ranchi (Reg. no. 1104/GO/Re/S/07/CPCSEA).

Inclusion criteria: Healthy male Wistar Albino rats weighing 150-200 gm.

Exclusion criteria: Unhealthy rats and weight <150 gm or >200 gm.

Drugs used: Diazepam 5 mg tablet, injection liraglutide 6 mg/ml vial, and donepezil tablet 5 mg. Liraglutide was the test drug whose effect was compared with donepezil. The dose of the drugs was calculated according to the body weight of Albino rats. All drugs were given i.p.

Drug preparation: Diazepam solution was prepared by dissolving 5 mg tablets of diazepam in 10 ml of normal saline. 0.2 ml of the freshly prepared solution was i.p. to produce amnesia (dose 0.1 mg/kg). Similarly, a 5 mg donepezil tablet was dissolved in 10 ml of normal saline and 0.2 ml of this solution was administered i.p. as the test drug. One ml of Liraglutide was dissolved in 5 ml of normal saline and 0.1 ml of this solution was administered i.p.

Instrument used: Morris water maze.

Principle

After giving training to the rats to locate the platform if amnesia is produced, the escape latency period increases, and then subsequent treatment with an anti-amnesic drug, the escape latency period returns to normal. Escape latency is the time (in seconds) to locate the hidden platform by rats.

Procedure

Group A rats were normal non-amnesic rats and group B, C, and D rats were made amnesic by administering 0.1 mg/kg of diazepam. All the rats were trained for one week to swim over water in a circular tank of MWM to locate the hidden platform. On the day of the experiment, the rats were placed in water at a designated starting location, and escape latency was noted. Immediately after administering diazepam, group B, C, and D rats were given normal saline, liraglutide, and donepezil, respectively, as treatment. Group A rats received only normal saline as shown in Table 1.

**Table 1:** Details of drugs administered to all groups of rats with their doses

GROUPS	NO. OF RATS	DIAZEPAM	DRUGS	DOSE
Negative control (A)	5		Normal saline	2 ml/kg
Positive control (B)	5	0.1mg/kg	Normal saline	2 ml/kg
Liraglutide (C)	5	0.1mg/kg	Liraglutide	0.1 mg/kg
Donepezil (D)	5	0.1mg/kg	Donepezil	0.1 mg/kg

After giving treatment, all rats were examined for their escape latency period after one, two, three, and four hours of test drug administration. Baseline escape latency was noted at 0 hours. On the same day of the next week, the same experiment was repeated and escape latency was noted for a total of five weeks to evaluate the retention of past events (memory).

Statistical analysis

The whole data was entered in a Microsoft Excel sheet (Microsoft Corp., Redmond, WA) and was analyzed using SPSS software version 25.0 (IBM Corp., Armonk, NY). The dependent variable was expressed as a mean with standard deviation. Data were analyzed for p-value using Tukey’s honestly significant difference (HSD) post hoc test and analysis of variance (ANOVA).

## Results

The escape latency period of rats in group C is shown in Table 2.

**Table 2:** Mean escape latency period (in seconds) of group C rats at 0, 1, 2, 3, and 4 hours after liraglutide administration SD - standard deviation

No. of Weeks	0 hour		1 hour		2 hour		3 hour		4 hour	
	mean	SD	mean	SD	mean	SD	mean	SD	Mean	SD
First week	45.6	11.18	69.8	9.97	65.6	10.97	63.5	9.9	61.14	6.2
Second week	46.8	14.42	68.4	15.28	63.6	13.54	59.4	9.38	56.8	7.62
Third week	44.2	5.92	69.9	5	63.9	4.95	56.4	3.07	53.7	3.29
Fourth week	45.8	9.86	66.65	8.69	55.3	9.29	51.6	9.15	50.9	6.27
Fifth week	44.9	13.17	66.2	13.02	54.9	12.13	50.1	80.7	48.2	8.14

The escape latency of rats of group D is shown in Table 3.

**Table 3:** Mean escape latency period (in seconds) of group D rats at 0, 1, 2, 3, and 4 hours after donepezil administration hr - hour; SD - standard deviation

No. of Weeks	0 hr (baseline)		1 hr		2 hr		3 hr		4 hr	
	mean	SD	mean	SD	mean	SD	mean	SD	Mean	SD
First week	45.8	10.9	71.4	7.72	66.1	5.33	61	5.73	58.3	4.78
Second week	46.4	12.85	67	10.57	61.7	10.81	57.1	4.59	54.1	5.21
Third week	46.15	10.97	67.7	11.13	58.35	6.88	55.9	6.82	52.8	3.44
Fourth week	45.95	9.24	69.35	9.3	56.35	8.3	53.15	5.47	51.8	1.46
Fifth week	46.6	13.17	67.25	10.33	53.25	9.39	50.25	4.48	48.7	3.85

The mean difference in latency periods between groups B and C and their significant level is shown in Table 4, as analyzed by Tukey’s HSD post-hoc test.

**Table 4:** Mean difference in escape latency (in seconds) between groups B and C and their significance level after analysis of data with one-way ANOVA followed by Tukey's HSD post-hoc test HSD - honestly significant difference; ANOVA - analysis of variance

No. of Weeks	2^nd^ hour		3rd hour		4^th^ hour	
	Mean difference	Significance level	Mean difference	Significance level	Mean difference	Significance level
First week	6.2	0.363	7.3	0.072	6.9	0.102
Second week	6.8	0.076	7.1	0.08	10.0	0.04
Third week	5.4	0.42	12.5	0.021	12.4	0.012
Fourth week	17.65	0.001	19.75	0.012	19.65	0.001
Fifth week	15.6	0.001	18.6	0.001	21.15	0.001

The mean difference in latency period between groups C and D and their significant level is shown in Table 5 as analyzed by Tukey’s HSD post-hoc test.

**Table 5:** Mean difference in escape latency (in seconds) between groups C and D and their significance level after analysis of data with one-way ANOVA followed by Tukey's HSD post-hoc test HSD - honestly significant difference; ANOVA - analysis of variance

No. of Weeks	2^nd^ hour		3^rd^ hour		4^th^ hour	
	Mean difference	Significant level	Mean difference	Significant level	Mean difference	Significant level
First week	0.5	0.72	2.5	0.060	3.1	0.12
Second week	1.9	0.6	2.3	0.070	2.7	0.20
Third week	0.55	0.08	0.5	0.9	0.9	0.08
Fourth week	1.05	0.067	1.55	0.063	0.9	0.082
Fifth week	1.65	0.062	0.15	0.062	0.5	0.56

Group A rats were used to compare whether group B rats became amnesic or not after the administration of diazepam. The induction of amnesia was indicated by an increase in escape latency. Group A rats did not show any increase in escape latency because they were given only normal saline. Liraglutide was found to possess significant anti-amnesic properties when compared to group B rats (positive control), which received only normal saline as treatment. Our data clearly suggest that both liraglutide and donepezil have anti-amnesic activity. The difference in escape latency became significant in the fourth hour in the second week and in the third hour in the third week, and in the fourth and fifth weeks, it was significant in the second, third, and fourth hours of drug administration as shown by a p-value of <0.05. However, when group C was compared to group D, there was no significant difference (p-value>0.05).

## Discussion

In this study, the learning and memory-enhancing property of liraglutide was explored and compared with the standard drug donepezil by using the MWM experimental model. Amnesia was produced by administering diazepam, a benzodiazepine (BZP) that produces amnesia as a common side effect [12]. We used escape latency for determining the anti-amnesic property of the test drug. If there was a decrease in escape latency, the drug was more potent, and vice versa. Our study findings showed that liraglutide has anti-amnesic properties. One experiment on mice also proves the neurotrophic and neuroprotective action of liraglutide [13]. Further, its effect is shown against hippocampal neuro-degeneration, which was studied among streptozotocin (STZ)-induced diabetic rats. Liraglutide also works by stimulating the anti-apoptotic pathways, so it also reduces the harmful effects of free radicals [14]. Patients of DM are at increased risk of cognition impairment in the initial stages and later on, there is more chance of the development of dementia. In animal models, GLP 1 analogs show neuroprotective effects, especially in the form of memory-enhancing drugs. Liraglutide slows down any further decline of memory in diabetic patients in the early stages [15]. Here too, learning and memory were improved by liraglutide. Pretreatment with liraglutide also decreased neuronal and synaptic damage [16].

MWM was used to assess spatial learning and memory in mice in one experiment. Here, liraglutide decreased the level of tau protein and NFTs in mice and decreased the number of neurodegenerative neurons in the hippocampus and cortex [17]. Insulin desensitization is one of the causes of AD along with demyelination, oxidative stress, and neuroinflammation. Impaired insulin signaling is associated with amyloid β and α synuclein deposition. GLP 1 receptor agonists are the potential target for such neurodegenerative diseases. They act by decreasing oxidative stress and cytokine production. They reduce the deposition of abnormal protein by crossing BBB [18]. So liraglutide may be used for patients with cognition impairment or even AD.

Rationale and novelty

Anticholinesterases are used in dementia. They work by increasing Ach levels in the brain, but they are not curative. They only slow the progress of neurodegeneration, thereby the cognition decline process becomes slow or is halted for a short time but is not prevented. Besides this, the peripheral side effects also appear with an increase in dose. So scientists are searching for drugs that work by another pathway and could enhance the cognitive potential of donepezil. Liraglutide is an anti-diabetic drug that works by decreasing apoptosis, oxidative stress, and neuroinflammation. So, it can be used as an add-on drug for dementia.

Limitations of the study

This model is effective for amnesic rats only, so its results cannot be projected for nondiabetic AD patients. Also, the present sample size is very small, so a large sample size could have better results. Further long-term studies are required to know the long-term effect of liraglutide as a memory enhancer drug and to evaluate its side effects or adverse effects if they occur. Besides this, we could not perform any brain site pathological studies that would have provided better evidence, but it should be considered in future research on this topic in rat models.

## Conclusions

From the findings of this study, we conclude that liraglutide has anti-amnesic properties. Donepezil, which is the standard drug for AD in which there is senile dementia, works by enhancing Ach levels in the brain. But liraglutide, which is a GLP-1 analog used in type 2 DM, can be used as an add-on drug because its mechanism of action is different from anticholinesterases like donepezil. Due to the adverse effects of donepezil in higher doses, the addition of liraglutide will reduce adverse events and increase the efficacy of donepezil.
